# An overview of mammalian p38 mitogen-activated protein kinases, central regulators of cell stress and receptor signaling

**DOI:** 10.12688/f1000research.22092.1

**Published:** 2020-06-29

**Authors:** Jiahuai Han, Jianfeng Wu, John Silke

**Affiliations:** 1 State Key Laboratory of Cellular Stress Biology, Innovation Center for Cell Biology, School of Life Sciences, Xiamen University, Xiamen, Fujian, 361005, China; 2 The Walter and Eliza Hall Institute, IG Royal Parade, Parkville, Victoria, 3052, Australia; 3 Department of Medical Biology, University of Melbourne, Parkville, Victoria, 3050, Australia

**Keywords:** p38, MAPK, inflammation, signalling

## Abstract

The p38 family is a highly evolutionarily conserved group of mitogen-activated protein kinases (MAPKs) that is involved in and helps co-ordinate cellular responses to nearly all stressful stimuli. This review provides a succinct summary of multiple aspects of the biology, role, and substrates of the mammalian family of p38 kinases. Since p38 activity is implicated in inflammatory and other diseases, we also discuss the clinical implications and pharmaceutical approaches to inhibit p38.

## p38 mitogen-activated protein kinases

p38α (originally named p38) was identified and cloned as a 38 kDa protein that was tyrosine-phosphorylated in response to LPS stimulation in mammalian cells ^1, 2^. Sequence comparison, on the day p38α was cloned, revealed that it belonged to the mitogen-activated protein kinase (MAPK) family and that a *Saccharomyces cerevisiae* osmotic response protein kinase HOG1 was a p38α homologue ^3– 5^. p38α was also named cytokine suppressive drug binding protein (CSBP) because it was identified as the target of a series of anti-inflammatory pyridinyl-imidazole compounds and as reactivating kinase (RK) because it phosphorylated and activated MK2 ^3– 5^. There are four members of the p38 group of MAPKs encoded by four different genes in mammals: p38α ( *MAPK14*, chromosome 6p21.31 in humans), p38β ( *MAPK11*, SAPK2b, Chr22q13.33) ^6^, p38γ ( *MAPK12*, ERK6, SAPK3, Chr22q13.33) ^7, 8^, and p38δ ( *MAPK13*, SAPK4, Serk4, Chr6p21.31) ^9, 10^. As can be surmised from their chromosomal locations, *MAPK14*/p38α and *MAPK13*/p38δ are physically close and separated by just over 15 kb, as are *MAPK12*/p38β and *MAPK11*/p38γ, which are separated by less than 2 kb. All the p38s contain a conserved Thr–Gly–Tyr (TGY) dual phosphorylation motif within the kinase activation loop, and both Thr and Tyr phosphorylation are necessary to fully activate the kinase ^11^. However, monophosphorylated p38α Thr ^180^ has some kinase activity *in vitro*, but a different substrate specificity, when compared with dual-site phosphorylated p38α ^12^. p38 group members are expressed ubiquitously, but p38γ and p38δ are enriched in certain cell types and tissues, such as p38γ in skeletal muscle and p38δ in the salivary, pituitary, and adrenal glands ^13^. p38β shares more amino acid sequence identity with p38α (~70%), while p38γ and p38δ share ~60% identity with p38α. p38γ and p38δ also share high sequence homology with cyclin-dependent kinases (CDKs) and are sensitive to some CDK inhibitors ^14^.

## Activation and inactivation of p38

p38α is involved in the response to almost all stressful stimuli, including LPS, UV light, heat shock, osmotic shock, inflammatory cytokines, T cell receptor ligation, glucose starvation, and oncogene activation ^2, 4, 5, 15– 20^. Under certain circumstances, it is also activated upon growth factor stimulation. It should be noted that the activation of p38 in some cases is cell type specific, since an activating stimulus in one cell type may inhibit p38 in other cell types ^21^. The study of p38 group members other than p38α has been less intensive; however, where it has been examined, the other p38s are frequently co-activated with p38α ^22^.

Like other MAPK signaling pathways, the activation of all p38s is mediated by a kinase cascade: MAPKKK (MAP3K), which activates MAPKK (MAP2K), which in turn activates MAPK. The MAP2K kinases MKK3 and MKK6 are the major upstream kinases for p38 activation ^23– 25^. Although MKK3 and MKK6 phosphorylate most p38 isoforms *in vitro*, selective activation and substrate specificity have been observed *in vivo* ^26^. MKK4 has also been reported to phosphorylate p38α and p38δ in specific cell types ^9^. A number of MAP3Ks have been reported to participate in p38 activation including TAK1 ^27^, ASK1 ^28^, DLK ^29^, and MEKK4 ^29, 30^. Low-molecular-weight GTP-binding proteins in the Rho family, such as Rac1 and Cdc42, can activate p38 through binding to MEK1 or MLK1, which function as upstream activators of MAP3K ^31, 32^.

p38α can also be activated by MAP2K-independent mechanisms. TAB1 (TAK1-binding protein 1) directly interacts with p38α and can promote trans autophosphorylation on Thr ^180^ and Tyr ^182^ and thus full activation of p38α ^33^. A subsequent study revealed that autophosphorylation of Thr ^180^ and Tyr ^182^ requires a conserved Thr ^185^ residue ^34^. TAB1-dependent p38α activation has been implicated in ischemic myocardial injury and T cell anergy ^35, 36^. TAB1 is also claimed to play a role in Sestrin-mediated p38α activation ^12^. Another MAP2K-independent activation is mediated by ZAP70 after T cell receptor ligation. ZAP70 can directly phosphorylate p38α/β on Tyr ^323^ ^18^, leading to autophosphorylation on Thr ^180^, one of the dual phosphorylation sites. As discussed, mono-Thr ^180^ phosphorylated p38 still has some kinase activity ^37^, and loss of ZAP70-mediated p38 activation in p38αβ ^Y323F^ double knock-in mice reduces autoimmunity and inflammation in several autoimmune disease models ^38– 40^. Interestingly, p38α also phosphorylates ZAP70, resulting in a decrease in the size and persistence of the T cell receptor signaling complex, and therefore acts as a feedback regulator of ZAP70 ^41^.

**Figure 1.**
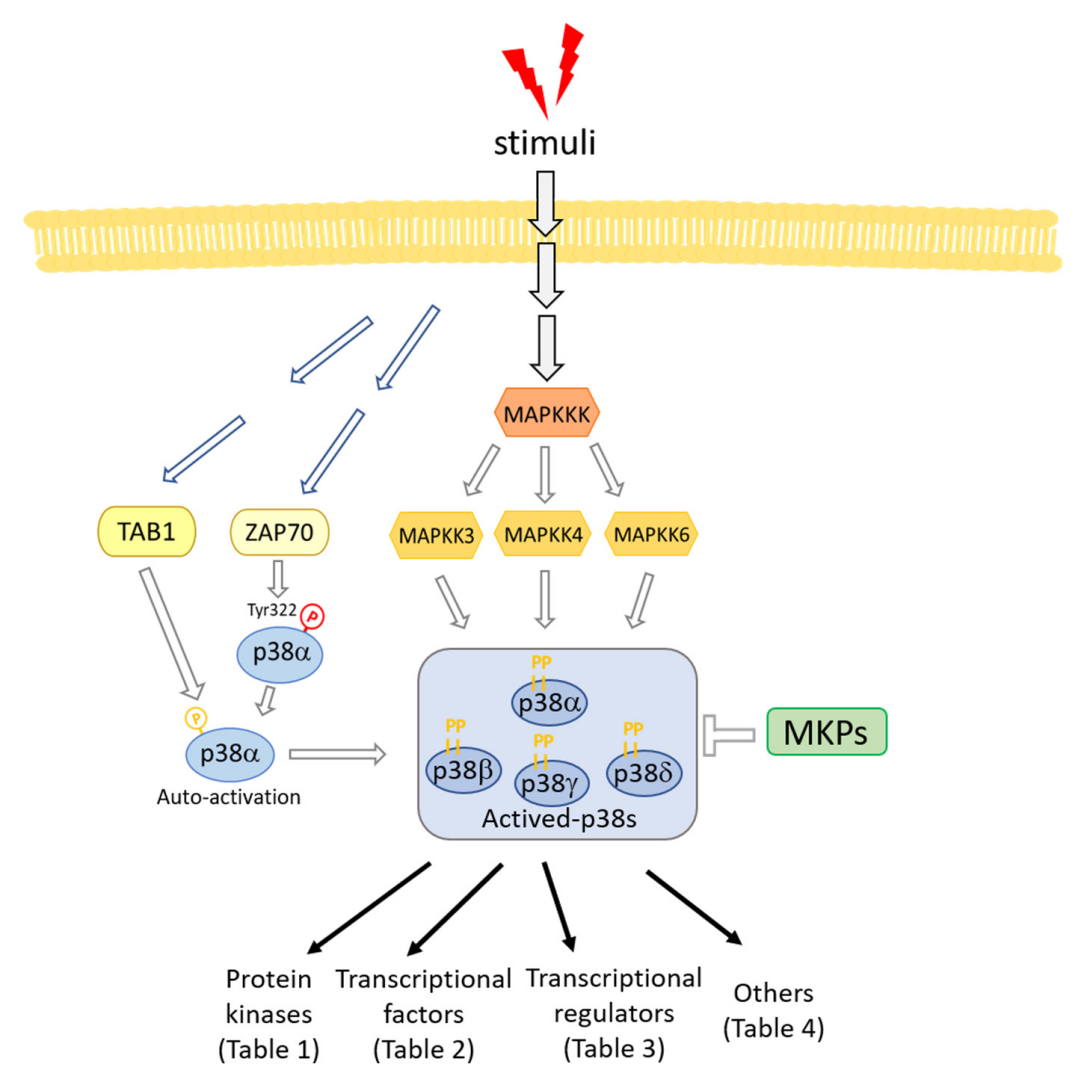
A diagram of the p38 pathway. MKP, mitogen-activated protein kinase phosphatase; TAB1, TAK1-binding protein 1; Tyr, tyrosine.

Conversely, de-phosphorylation of both threonine and tyrosine residues in the activation loop inactivates MAPKs, and this is mainly carried out by dual-specificity phosphatases of the MAPK phosphatase (MKP)/dual specificity phosphatase (DUSP) family ^42^. Although several MKPs have been reported to dephosphorylate p38α, MKP1/DUSP1, MKP5/DUSP10, MKP8/DUSP26, and DUSP8 are more potent inhibitors of p38α and JNK than ERK ^43^. A recent report showed that DUSP12 is also a p38α phosphatase ^44^. While there are a number of p38α DUSPs, no DUSP for p38γ or p38δ has been reported, and these two p38s are resistant to several known p38α MKPs such as MKP1, 3, 5, and 7 ^45^. p38α-dependent upregulation of MKP1 was reported and is believed to be part of a negative feedback loop of p38α activation ^46^. Other types of phosphatases have also been reported to target p38 MAPKs, such as CacyBP/SIP ^47^, Wip1 ^48^, and PP2C ^49, 50^. The substrate specificity between p38 and phosphatases and the related physiological functions *in vivo* still need further investigation. p38γ has also been reported to be degraded by a p38/JNK/ubiquitin-proteasome-dependent pathway, which represents an additional mechanism by which p38 kinases may cross regulate each other ^51^. Yet other ways of regulating p38 are suggested from studies in *Caenorhabditis elegans*, where a genetic screen for resistance against bacterial infection identified RIOK-1, an atypical serine kinase and human RIO kinase homolog, as a suppressor of the p38 pathway ^52^. As RIOK-1 is a transcriptional target of the p38 pathway in *C. elegans*, this suggests that RIOK-1 is part of a negative feedback loop. A brief summary of the p38 pathway is shown in Figure 1.

## Downstream substrates of p38

### Protein kinases

The p38 MAPK cascade does not end at p38. Members of the MAPK-activated protein kinase (MAPKAPK) family such as MK2, MK3, and MK5 (PRAK) are all p38 substrates ^3, 4, 53– 55^. The MKs have a broad range of substrates that extend the range of functions regulated by p38 kinases. Mitogen- and stress-activated protein kinase-1/2 (MSK1/2), which are important for CREB activation and chromosome remodeling, have also been identified as substrates of p38α ^56^. MNK1/2, kinases that phosphorylate the eukaryotic initiation factor-4e (eIF-4E), are phosphorylated by p38α ^57, 58^. p38α has also been reported to inactivate murine GSK3β by phosphorylating Ser ^389^, and since GSK3β is required for the continuous degradation of β-catenin in the Wnt signaling pathway, this can lead to an accumulation of β-catenin ^59, 60^. It was also reported that p38δ negatively regulates insulin secretion by catalyzing an inhibitory phosphorylation of PKD1 ^61^. A number of p38 protein kinase substrates are summarized in Table 1.

**Table 1.** Substrates of p38 group members – kinases.

Substrate	Kinase	Function	References
MAPKAPK2 (MK2)	p38α, p38β, p38γ, p38δ	Activates the kinase substrate	Freshney NW *et al*., *Cell,* 1994 ^4^ Rouse J *et al*., *Cell,* 1994 ^3^
MAPKAPK3 (MK3)	p38α, p38β, p38γ, p38δ	Activates the kinase substrate	McLaughlin MM *et al*., *J Biol Chem,* 1996 ^54^
MNK1/2	p38α	Activates the kinase substrate	Fukunaga R *et al., EMBO J,* 1997 ^58^ Waskiewicz AJ *et al., EMBO J,* 1997 ^57^
MSK1/2	p38α	Activates the kinase substrate	Deak M *et al., EMBO J,* 1998 ^56^ Pierrat B *et al., J Biol Chem,* 1998 ^77^
PAK6	p38α	Activates the kinase substrate	Kaur R *et al., J Biol Chem,* 2005 ^78^
PIP4Kb	p38α	Inactivates the kinase substrate	Jones DR *et al., Mol Cell,* 2006 ^79^
RPAK (MK5)	p38α, p38β	Activates the kinase substrate	New L *et al., EMBO J,* 1998 ^55^
PKCε	p38α, p38β	Completes cytokinesis	Saurin AT *et al., Nat Cell Biol,* 2008 ^80^
GSK3β	p38α	Inactivates the kinase substrate, activates Wnt pathway.	Bikkavilli RK *et al., J Cell Sci,* 2008 ^60^ Thornton TM *et al., Science,* 2008 ^59^

GSK3β, glycogen synthase kinase 3 beta; MAPKAPK, mitogen-activated protein kinase activated protein kinase; MSK1/2, mitogen- and stress-activated protein kinase; PAK6, p21-activated kinase 6; PIP4Kb, phosphatidylinositol 5 phosphate 4-kinase; PKCε, protein kinase C epsilon type.

### Transcription factors

p38 targets a large number of transcription factors, including myocyte-specific enhancer factor 2 (MEF2) family members, cyclic AMP-dependent transcription factor 1, 2, and 6 (ATF-1/2/6), CHOP (growth arrest and DNA damage inducible gene 153, or GADD153), p53, C/EBPβ, MITF1, DDIT3, ELK1/4, NFAT, and STAT1/4. p38 phosphorylation of transcription factors predominantly leads to enhanced transcriptional activity. However, in some cases, it represses transcription, and this is summarized in Table 2. Transcription factor phosphorylation by p38 is often stimulus and cell type dependent and plays a role in the cellular response to inflammation, DNA damage, metabolic stress, and many other stresses ^62– 76^. The effects of p38 on transcription seem to constitute the major part of p38’s responses to stress stimuli.

**Table 2.** Substrates of p38 group members – transcription factors.

Substrate	Kinase	Function	References
ATF2	p38α, p38β, p38γ, p38δ	Enhances transcriptional activity	Cuenda A *et al*., *EMBO J,* 1997 ^81^ Jiang Y *et al*., *J Biol Chem,* 1997 ^9^
C/EBPα	p38α	Enhances transcriptional activity	Qiao L *et al*., *J Biol Chem,* 2006 ^82^
C/EBPβ	p38α	Enhances transcriptional activity	Engelman JA *et al*., *J Biol Chem,* 1998 ^83^
C/EBPε	p38α	Enhances transcriptional activity	Williamson EA *et al*., *Blood,* 2005 ^84^
CHOP	p38α, p38β	Enhances transcriptional activity	Wang XZ *et al*., *Science,* 1996 ^68^
E2F4	p38α	Enhances transcriptional activity	Morillo SM *et al*., *Mol Cell Biol,* 2012 ^85^
Elk-1	p38α	Enhances transcriptional activity in specific cell types	Janknecht R *et al*., *EMBO J,* 1997 ^67^ Whitmarsh AJ *et al*., *Mol Cell Biol,* 1997 ^66^
ERα	p38α	Enhances nuclear localization and transcriptional activity	Lee H *et al*., *Mol Cell Biol,* 2002 ^86^
Fos	p38α, p38β, p38γ, p38δ	Enhances transcriptional activity	Tanos T *et al*., *J Biol Chem,* 2005 ^87^
FOXO3a	p38α	Enhances nuclear relocalization	Ho KK *et al., J Biol Chem,* 2012 ^88^
GR	p38α	Enhances transcriptional activity	Miller AL *et al., Mol Endocrinol,* 2005 ^89^
IUF1	p38α, p38β	Enhances transcriptional activity	Macfarlane WM *et al., J Biol Chem,* 1997 ^90^
JDP2	p38α	N/D	Katz S *et al., Biochem J,* 2002 ^91^
c-JUN	p38α, p38β, p38γ	Enhances transcriptional activity	Humar M *et al., Int J Biochem Cell* *Biol,* 2007 ^92^
MafA	p38α, p38β, p38γ, p38δ	Enhances transcriptional activity	Sii-Felice K *et al., FEBS Lett,* 2005 ^93^
MEF2A	p38α, p38β, p38δ	Enhances transcriptional activity	Zhao M *et al., Mol Cell Biol,* 1999 ^94^
MEF2C	p38α, p38β p38γ, p38δ	Enhances transcriptional activity	Han J *et al., Nature,* 1997 ^62^
MEF2D	p38α	Enhances recruitment of Ash2L to muscle-specific promoters	Zhao M *et al., Mol Cell Biol,* 1999 ^94^ Rampalli S *et al., Nat Struct Mol Biol,* 2007 ^73^
MITF	p38α	Enhances transcriptional activity	Mansky KC *et al., J Biol Chem,* 2002 ^95^
MRF4	p38α	Represses transcriptional activity	Suelves M *et al., EMBO J,* 2004 ^96^
NFATc1	p38α	Enhances transcriptional activity and interaction with PU.1	Matsumoto M *et al., J Biol Chem,* 2004 ^97^
NFATc4	p38α, p38β p38γ	Represses nuclear localization and transcriptional activity	Yang TT *et al., Mol Cell Biol,* 2002 ^98^
NR4A	p38α	Enhances transcriptional activity	Sekine Y *et al., J Cell Sci,* 2011 ^99^
Nur77	p38α	Disrupts interaction with p65 and represses transcriptional activity	Li L *et al., Nat Chem Biol,* 2015 ^100^
Osterix	p38α	Enhances recruitment of coactivators	Ortuño MJ *et al., J Biol Chem,* 2010 ^101^
p53	p38α	Increases protein stability and apoptosis	Bulavin DV *et al., EMBO J,* 1999 ^69^
Pax6	p38α	Enhances transcriptional activity	Mikkola I *et al., J Biol Chem,* 1999 ^102^
PPARα	p38α	Enhances transcriptional activity	Barger PM *et al., J Biol Chem,* 2001 ^103^
SAP1	p38α, p38β p38γ, p38δ	Enhances transcriptional activity	Janknecht R *et al*., *EMBO J,* 1997 ^67^
Smad3	p38α	Enhances nuclear translocation	Hayes SA *et al*., *Oncogene,* 2003 ^104^
Snail	p38α	Increases protein stability and transcriptional activity	Ryu KJ *et al*., *Cancer Res,* 2019 ^105^
STAT1	p38α, p38β	Enhances transcriptional activity	Kovarik P *et al*., *Proc Natl Acad Sci* *U S A,* 1999 ^106^
STAT4	p38α	Enhances transcriptional activity	Visconti R *et al*., *Blood,* 2000 ^107^
TEAD4	p38α	Enhances cytoplasmic translocation and suppresses transcriptional activity	Lin KC *et al*., *Nat Cell Biol,* 2017 ^76^
Twist1	p38α	Increases protein stability and transcriptional activity	Hong J *et al*., *Cancer Res,* 2011 ^108^
USF1	p38α	Enhances transcriptional activity	Galibert MD *et al*., *EMBO J,* 2001 ^71^
Xbp1s	p38α	Enhances nuclear translocation and transcriptional activity	Lee J *et al*. *, Nat Med,* 2011 ^75^

ATF2, activating transcription factor 2; C/EBP, CCAAT/enhancer binding protein; CHOP, CCAAT/enhancer-binding protein homologous protein; ER, estrogen receptor; GR, glucocorticoid receptor; IUF1, insulin upstream factor 1; JDP2, Jun dimerization protein 2; MEF, myocyte-specific enhancer factor; MITF, microphthalmia transcription factor; MRF, muscle regulatory factor; NFAT, nuclear factor of activated T cells; Pax6, paired box 6; PPARα, peroxisome proliferator-activated receptor alpha; TEAD4, TEA domain family transcription factor 4; USF1, upstream transcription factor 1; Xbp1s, spliced form of X-box binding protein 1.

### Transcriptional regulators

A large number of transcriptional regulators, including epigenetic enzymes, are substrates of p38, and these are summarized in Table 3. The SWI–SNF complex subunit BAF60 is phosphorylated and inactivated by p38 during skeletal myogenesis ^109, 110^, and EZH2, the catalytic component of the Polycomb Repressive Complex 2 (PRC2), was also found to be phosphorylated by p38, particularly in ER-negative breast cancer samples ^111^. Besides its transcriptional function, dATF-2 is also involved in heterochromatin formation, and stress-induced phosphorylation of dATF-2 by p38 disrupts heterochromatin in *Drosophila* ^112^.

**Table 3.** Substrates of p38 group members – transcriptional regulators.

	Substrate	Kinase	Function	References
Chromatin remodeling regulators	BAF60c	p38α, p38β	Activates transcription of MyoD- target genes	Simone C *et al*., *Nat Genet,* 2004 ^109^ Forcales SV *et al*., *EMBO J,* 2012 ^110^
	RNF2	p38α	Modulates gene expression and histone 2B acetylation	Rao PS *et al*., *Proteomics,* 2009 ^124^
	EZH2	p38α	Promotes cytoplasmic localization	Anwar T *et al*., *Nat Commun,* 2018 ^111^
	dAFF2	p38α, p38β	Disrupts heterochromatin formation	Seong K-H *et al*., *Cell,* 2011 ^112^
Other regulators	CRTC2	p38α	Enhances nucleocytoplasmic transport and represses transcription activity	Ma H *et al*., *Mol Cell Biol,* 2019 ^125^
	E47	p38α, p38β	Enhances the formation of MyoD/ E47 heterodimers	Page JL *et al., J Biol Chem,* . 2004 ^126^ Lluís F *et al*., *EMBO J,* 2005 ^127^
	HBP1	p38α	Increases protein stability and represses transcription	Xiu M *et al*., *Mol Cell Biol,* 2003 ^128^
	p18(Hamlet)	p38α, p38β	Increases protein stability and enhances transcription	Cuadrado A *et al*., *EMBO J,* 2007 ^129^
	PGC-1α	p38α, p38β	Increases protein stability and enhances transcription	Puigserver P *et al., Mol Cell,* 2001 ^130^
	Rb1	p38α, p38γ	Induces Rb degradation and cell death; suppresses Rb activity and promotes the G0-to-G1 transition	Delston RB *et al., Oncogene,* 2011 ^131^ Tomás-Loba A *et al., Nature,* 2019 ^14^
	SRC-3	p38α	Induces SRC-3 degradation and suppresses RARα-dependent transcription	Giannì M *et al., EMBO J,* 2006 ^132^

CRTC2, CREB-regulated transcription coactivator 2; HBP1, HMG-box transcription factor 1; PGC-1α, peroxisome proliferator-activated receptor gamma co-activator 1 alpha; RAR, retinoic acid receptor; RNF2, ring finger protein 2.

### Other substrates

Given the wide range of responses that p38 is involved in, it is not surprising that many p38 substrates cannot be so easily categorized into groups, and these miscellaneous substrates are summarized in Table 4. Some of them are involved in metabolism such as Raptor phosphorylation by p38β, which enhances mTORC1 activity in response to arsenite-stress ^113^, and DEPTOR (mTOR-inhibitory protein) phosphorylation by p38γ and p38δ, leading to its degradation and mTOR hyperactivation ^114^. p38α phosphorylation of Tip60 at Thr ^158^ promotes senescence and DNA-damage-induced apoptosis ^115, 116^. Some p38 substrates are cell death regulators. In the ER stress response, p38α locates to the lysosome and phosphorylates the chaperone-mediated autophagy (CMA) receptor LAMP2A, leading to activation of CMA and thus protecting cells from ER stress-induced death ^117^.

**Table 4.** Substrates of p38 group members – others.

	Substrate	Kinase	Function	References
Cell-cycle regulators	Cdc25A	p38α	Increases protein stability	Goloudina A *et al*. *, Cell Cycle,* 2003 ^133^
	Cdc25B	p38α	Increases protein stability	Lemaire M *et al*., *Cell Cycle,* 2006 ^134^
	Cyclin D1	p38α	Causes ubiquitination and degradation of cyclin D1	Casanovas O *et al*., J Biol Chem *,* 2000 ^135^
	Cyclin D3	p38α, p38β p38γ, p38δ	Causes ubiquitination and degradation of cyclin D3	Casanovas O *et al*., *Oncogene,* 2004 ^136^
	p57kip2	p38α	Enhances interaction with CDKs and inhibits CDKs	Joaquin M *et al*., *EMBO J,* 2012 ^137^
Cell-death regulators	Bax	p38α	Prevents Bcl-2–Bax heterodimer formation, enhances apoptosis	Min H *et al*., *Mol Carcinog,* 2012 ^138^
	BimEL	p38α	Enhances apoptosis	Cai B *et al*., *J Biol Chem,* 2006 ^139^
	Caspase-3	p38α	Inhibits caspase-3 activity and apoptosis	Alvarado-Kristensson M *et al*., *J Exp Med,* 2004 ^140^
	Caspase-8	p38α	Inhibits caspase-8 activity and apoptosis	Alvarado-Kristensson M *et al*., *J Exp Med,* 2004 ^140^
	Caspase-9	p38α	Inhibits caspase-9 activity and apoptosis	Seifert A *et al*., *Cell Signal,* 2009 ^141^
DNA/RNA binding proteins	Cdt1	p38α, p38β	Increases protein stability	Chandrasekaran S *et al*. *, Mol Cell Biol,* 2011 ^142^
	Drosha	p38α	Enhances nuclear export and degradation	Yang Q *et al*. *, Mol Cell,* 2015 ^143^
	FBP2	p38α	Promotes prothrombin mRNA 3' end processing	Danckwardt S *et al*. *, Mol Cell,* 2011 ^144^
	FBP3	p38α	Promotes prothrombin mRNA 3' end processing	Danckwardt S *et al*. *, Mol Cell,* 2011 ^144^
	H2AX	p38α, p38β	Promotes serum starvation-induced apoptosis	Lu C *et al*. *, FEBS Lett,* 2008 ^145^
	H3	p38α	N/D	Zhong SP *et al*. *, J Biol Chem,* 2000 ^146^
	HuR	p38α, p38β	Enhances cytoplasmic accumulation and increases mRNA stability	Lafarga V *et al*. *, Mol Cell Biol,* 2009 ^147^
	KSRP	p38α, p38β	Prevents KSRP-mediated ARE-directed mRNA decay	Briata P *et al*. *, Mol Cell,* 2005 ^148^
	Rps27	p38α	N/D	Knight JD *et al*., *Skelet Muscle,* 2012 ^149^
	SPF45	p38α	Inhibits Fas alternative splicing (exon 6 exclusion)	Al-Ayoubi AM *et al*., *Mol Cell Biol,* 2012 ^150^
Endocytosis regulators	EEA1	p38α	Promotes recruitment to endocytic membranes and enhances MOR endocytosis	Macé G *et al*. *, EMBO J,* 2005 ^151^
	Rabenosyn-5	p38α	Promotes recruitment to endocytic membranes and enhances MOR endocytosis	Macé G *et al*. *, EMBO J,* 2005 ^151^
	GDI-2	p38α	Enhances GDI:Rab5 complex formation and modulates endocytosis	Cavalli V *et al*. *, Mol Cell,* 2001 ^152^
MAPK pathway regulator	JIP4	p38α	Enhances p38 activity	Kelkar N *et al*. *, Mol Cell Biol,* 2005 ^153^
	Tip60	p38α	Enhances the pro-senescent function of Tip60	Zheng H *et al*. *, Mol Cell,* 2013 ^115^
	TAB1	p38α	Inhibits TAK1 activity	Cheung PC *et al*. *, EMBO J,* 2003 ^154^
	TAB3	p38α	Inhibits TAK1 activity	Mendoza H *et al*. *, Biochem J,* 2008 ^155^
	FRS2	p38α	Downregulates FGF1-induced signaling	Zakrzewska M *et al*., *Int J Mol Sci,* 2019 ^156^
Membrane proteins	EGFR	p38α	Induces EGFR internalization	Winograd-Katz SE *et al*., *Oncogene,* 2006 ^157^
	FGFR1	p38α	Regulates translocation of exogenous FGF1 into the cytosol/nucleus	Sørensen V *et al*., *Mol Cell Biol,* 2008 ^158^
	Nav1.6	p38α	Promotes interaction with NEDD-4 and protein degradation	Gasser A *et al*., *J Biol Chem,* 2010 ^159^
	NHE1	p38α	Induces intracellular alkalinization	Khaled AR *et al*. *, Mol Cell Biol,* 2001 ^160^
	PLA2	p38α	N/D	Börsch-Haubold AG *et al*. *, J Biol Chem,* 1998 ^161^
	TACE	p38α, p38β	Increases TACE-mediated ectodomain shedding and TGF-alpha family ligand release	Xu P *et al*. *, Mol Cell,* 2010 ^162^
	ZAP70	p38α	Phosphorylation of ZAP70 increases stability of T cell receptor	Giardino Torchia ML *et al*. *, Proc Natl Acad Sci* *U S A,* 2018 ^41^
Structure proteins	Caldesmon	p38α	N/D	Hedges JC *et al*., *Am J Physiol,* 1998 ^163^
	Hsp27	p38α	N/D	Knight JD *et al*., *Skelet Muscle,* 2012 ^149^
	Keratin 8	p38α	Regulates cellular keratin filament reorganization	Ku NO *et al*., *J Biol Chem,* 2002 ^164^
	Lamin B1	p38α	Enhances lamin B1 accumulation	Barascu A *et al*., *EMBO J,* 2012 ^165^
	Paxillin	p38α	Required for NGF-induced neurite extension of PC-12 cells	Huang C *et al*., *J Cell Biol,* 2004 ^166^
	Stathmin	p38δ	N/D	Parker CG *et al*., *Biochem Biophys Res* *Commun,* 1998 ^167^
	SAP97	p38γ	Modulating the association of this protein with other cytoskeleton proteins	Sabio G *et al*., *EMBO J,* 2005 ^168^
	Tau	p38α, p38γ, p38δ	Enhances formation of paired helical filaments Inhibits amyloid-β toxicity in Alzheimer's mice	Reynolds CH *et al*., *J Neurochem,*1997 ^169^ Ittner A *et al*., *Science,* 2016 ^170^
	Tensin1	p38α	Regulates the binding specificity of tensin1 to different proteins	Hall EH *et al*. *, Mol Cell Proteomics,* 2010 ^171^
Others	DEPTOR	p38γ, p38δ	Enhances degradation and mTOR hyperactivation	González-Terán B *et al*. *, Nat Commun,* 2016 ^114^
	GS	p38β	Required for subsequent phosphorylation to inhibit enzyme activity	Kuma Y *et al*. *, Biochem J,* 2004 ^172^
	LAMP2A	p38α	Activates chaperone-mediated autophagy	Li W *et al., Nat Commun,* 2017 ^117^
	Parkin	p38α	Decreases its interaction with PINK1 and suppresses mitophagy	Chen J *et al., Cell Death Dis,* 2018 ^173^
	p47 ^phox^	p38α	Promotes NADPH oxidase activation and superoxide production	Makni-Maalej K *et al*. *, J Immunol,* 2012 ^174^
	p62	p38γ, p38δ	Enhances mTORC1 activity	Linares JF *et al., Cell Rep,* 2015 ^175^ Koh A *et al., Cell,* 2018 ^176^
	Raptor	p38β	Enhances mTORC1 activity in response to arsenite stress	Wu X-N *et al*. *, J Biol Chem,* 2011 ^113^
	Rpn2	p38α	Inhibits proteasome activity	Lee SH *et al*. *, J Biol Chem,* 2010 ^177^
	Siah2	p38α	Increases Siah2-mediated degradation of PHD3	Khurana A *et al*. *, J Biol Chem,* 2006 ^178^

CDK, cyclin-dependent kinase; EGFR, epidermal growth factor receptor; FBP1, far upstream binding protein; FGF1, fibroblast growth factor 1; FGFR1, fibroblast growth factor receptor 1; FRS2, fibroblast growth factor receptor substrate 2; GDI, GDP dissociation inhibitor; KSRP, hnRNPK-homology type splicing regulatory protein; MAPK, mitogen-activated protein kinase; mTORC1, mammalian target of rapamycin complex 1; NADPH, nicotinamide adenine dinucleotide phosphate; NGF, nerve growth factor; NHE1, Na ^+^/H ^+^ exchanger isoform 1; PHD3, prolyl hydroxylase 3; PLA2, phospholipase A2; SAP97, synapse-associated protein 97; TAB, transforming growth factor-β-activated protein kinase-1-binding protein; TACE, tumor necrosis factor-alpha-converting enzyme; TAK1, transforming growth factor β-activated kinase 1; TGF, transforming growth factor.

## Biological functions of the p38 pathway

### Embryo development

p38α is required for embryo development, since the mouse *Mapk14* ^–/–^ embryo dies between embryonic days (E) 10.5 and 12.5 ^118– 121^. Mutant mice with a single Thr ^180^ to Ala mutation or with the double T180A Y182F mutation are also embryonic lethal ^122, 123^. Surprisingly, given the importance of the dual phosphorylation for complete p38 activation, substitution of Tyr ^182^ with Phe results in mice that have reduced p38 signaling but are nevertheless viable ^123^, although this is consistent with previous studies showing that the p38 phosphorylated on Thr ^180^ alone retains some activity *in vitro* ^37^. Histological analysis demonstrates that p38α is required for placental angiogenesis, but not embryonic cardiovascular development, and tetraploid rescue of the placental defect in *Mapk14* ^–/–^ embryos confirmed that p38α is essential for extraembryonic development ^120, 121^. Given the important role that p38 and MK2 plays in regulating TNF-induced cell death ^179– 182^, it is intriguing that the *Mapk14* ^–/–^ embryonic lethal phenotype is very similar to that observed in other mice with defects in the TNF death pathway. Caspase-8, FADD, and cFLIP knock-out mice also die at E10.5, and this is due to TNF-dependent endothelial cell death and disruption of the vasculature in the yolk sac ^183, 184^. Other p38 isoforms are not necessary for embryo development, but p38α and p38β have overlapping functions, as *Mapk14 ^loxp/loxp^Mapk11 ^–/–^Sox2-Cre* embryos die before E16.5 with spina bifida that correlates with neural hyperproliferation and increased apoptosis in the liver, which was not observed in *Mapk14* ^∆/∆^ *Sox2-Cre* embryos ^185^. Remarkably, p38α appears to have a very specific function during embryogenesis because when p38α was replaced by p38β in the *Mapk14* chromosomal locus, which thereby placed p38β under the control of the endogenous p38α promoter, it was unable to rescue the embryonic lethality induced by loss of p38α ^185^.

### Immune responses

p38 is activated by many inflammatory stimuli, and its activity is important for inflammatory responses. Macrophage-specific deletion of *Mapk14* inhibits inflammatory cytokine production and protects mice from CLP-induced sepsis ^186^. p38α controls the production of inflammatory cytokines, such as TNF and IL-6, at many levels. It directly phosphorylates transcription factors, such as MEF2C ^62, 186^, and regulators of mRNA stability, such as hnRNPK-homology (KH) type splicing regulatory protein (KSRP) ^187^. MEF2C appears to play an anti-inflammatory role in endothelial cells *in vivo* ^188^. Via MK2/MK3, p38 also upregulates cytokine mRNA transcription by the serum response transcription factor (SRF) ^189^, and similarly, via MK2/MK3, p38 regulates mRNA stability by phosphorylating and inactivating TTP/Zfp36, a protein that promotes rapid turnover of AU-rich mRNAs, many of which are cytokine mRNAs ^187, 190^. p38 activation also induces the expression of inflammatory mediators such as COX-2, MMP9, iNOS, and VCAM-1, which are involved in tissue remodeling and oxidation regulation ^191– 194^. The p38 pathway also regulates adaptive immunity. p38α participates in antigen processing in CD8 ^+^ cDCs ^195^, and ZAP70-mediated p38α/β activation is important for T cell homeostasis and function ^18^. In B cells, p38α is important for CD40-induced gene expression and proliferation of B cells ^196^, and the p38α–MEF2c axis is believed to be necessary for germinal center B (GCB) cell proliferation and survival ^197, 198^. Excessive activation of p38α has been observed in many inflammatory diseases, such as inflammatory bowel disease (IBD), asthma, rheumatoid arthritis, and steatohepatitis ^199– 201^. The other members of the p38 family also play roles in immune responses. For example, p38γ and p38δ are required for neutrophil migration to damaged liver in non-alcoholic fatty liver disease ^202^ and inhibition of eukaryotic elongation factor 2 in LPS-induced liver damage ^203^. p38δ is required for neutrophil accumulation in acute lung injury ^204^. These observations, and the role that p38s play in TNF production, led to enormous pharmaceutical efforts to develop p38 inhibitors to treat chronic inflammatory diseases. However, unfortunately, these drugs were not efficacious in these diseases ^205^.

### Cell cycle

p38 has been implicated in G1 and G2/M phases of the cell cycle in several studies. The addition of activated recombinant p38α caused mitotic arrest *in vitro*, and an inhibitor of p38α/β suppressed activation of the checkpoint by nocodazole in NIH3T3 cells ^206^. G1 arrest caused by Cdc42 overexpression is also dependent on p38α in NIH3T3 cells ^207^. Besides, p38γ is specially required for gamma-irradiation-induced G2 arrest ^208^. The link between p38 and cell cycle control has been proposed through the regulation of several p38 substrates. Both p38α and p38γ regulate cell cycle progression via Rb but in opposite directions ^14, 209^. HBP1 represses the expression of cell cycle regulatory genes during cell cycle arrest in a p38-dependent manner ^210^; p53 and p21 activation by p38α prevented G1 progression through blockade of CDK activity ^211, 212^. The p38 pathway is also involved in cell cycle progress, as it is essential for self-renewal of mouse male germline stem cells ^213^ and its regulation of G1-length plays a role in cell size uniformity ^214^.

### Cell differentiation

Participation of p38 in cell differentiation has been reported in certain cell types. p38α activity is essential for neuronal differentiation in PC-12 cells and EPO-induced differentiation in SKT6 cells ^20, 215^. Treatment of 3T3-L1 fibroblasts with specific p38α/β inhibitors prevents their differentiation into adipocytes by reducing C/EBPβ phosphorylation ^83^, and p38α-dependent phosphorylation of MEF2C and BAF60 is critical for myogenic differentiation ^110, 216^. Intestinal epithelial cell-specific deletion of p38α also influences goblet cell differentiation in a Notch-dependent manner ^200^.

### Cell metabolism

p38 group members participate in many cellular events related to metabolism. The p38β–PRAK axis specifically phosphorylates Rheb and suppresses mTORC1 activity under energy depletion conditions ^22^. DEPTOR, an inhibitor of mTORC, can be phosphorylated by p38γ and p38δ, leading to its degradation ^123^. Meanwhile, p38δ directly phosphorylated p62 to enhance mTORC1 activity in response to amino acids ^175^. In brown adipocytes, p38α functions as a central mediator in β-adrenergic-induced UCP1 expression ^217, 218^, while in white adipocytes, p38α inactivation leads to elevated white-to-beige adipocyte reprogramming and resistance to diet-induced obesity ^219, 220^. In hepatocytes, p38α controls lipolysis and protects against nutritional steatohepatitis. Thus, mice with hepatocyte-specific loss of p38α developed more severe steatohepatitis than wild type mice when fed high-fat or -cholesterol diets. Intriguingly, macrophage specific deletion of p38 had the opposite effect in the same high-fat diets and resulted in less steatohepatitis than in wild type mice, which probably reflects the inflammatory role of p38 in macrophages ^199^. p38α also directly phosphorylates Xbp1s to enhance its nuclear migration for maintaining glucose homeostasis in obesity ^75^. However, p38α also functions as a negative regulator of AMPK signaling in maintaining gluconeogenesis, and hepatic p38α could be a drug target for hyperglycemia ^221^. It was also reported that p38γ directly phosphorylated p62 under imidazole propionate stimulation to promote mTORC1 activity in hepatocytes ^176^. Interestingly, AMPK also triggers the recruitment of p38α to scaffold protein TAB1 for p38α autoactivation in human T cells ^222^.

### Cell senescence

p38α appears to play a pivotal role in senescence. Constitutive activation of the p38 pathway by active MKK3 or MKK6 induces senescence in several cell types ^223, 224^, and p38α activity is responsible for senescence induced by multiple stimuli, such as telomere shortening ^225, 226^, H _2_O _2_ exposure ^227, 228^, and chronic oncogene activation ^19, 223, 229^. p38α/β-specific inhibitors have been successfully used to prevent cellular senescence in cultivated human corneal endothelial cells ^230^. Since cellular senescence is considered a defense strategy against oncogene activation, the p38 pathway plays important roles in tumorigenesis ^231^. Meanwhile, p38α activity is important for senescence-associated secretory phenotype (SASP), and its inhibition markedly reduces the secretion of most SASP factors, suggesting multiple roles for the p38 pathway in senescence ^232– 235^.

### Cell survival and death

The role of the p38 pathway in cell fate is cell type and stimulus dependent. For example, p38α becomes activated upon NGF withdrawal in PC-12 cells, and p38α activated by overexpression of MKK3 induced apoptosis in NGF differentiated PC-12 cells ^211^. Similarly, inhibition of p38 with PD169316 blocked NGF withdrawal-induced apoptosis in PC-12 cells ^236, 237^. The interplay between the p38 pathway and caspases, the central regulators/executors of apoptosis, is complicated because p38α activity can be elevated in a caspase-dependent manner in death stimulus treated cells ^238, 239^, and caspase activity can also be elevated in MKK6E (dominant active form) overexpressed cells ^239, 240^. In contrast, inhibition of caspase-8 and caspase-3 by p38α-mediated phosphorylation in neutrophils was also reported ^140^. Recent studies show that p38-activated MK2 directly phosphorylates RIPK1 in TNF-treated cells or pathogen-infected cells, limiting TNF-induced cell death ^180– 182^. This represents an interesting link between cytokine production induced by TNF and cell death because TNF-induced MK2/MK3 phosphorylation of tristetraprolin/Zfp36 inactivates it and leads to increased stability of cytokine mRNAs ^190^. Aberrant p38α activity is observed in many tumor cells, and inhibition of p38α/β enhances cell death in these cells ^241, 242^.

## Perspectives

p38 is one of the most researched of all proteins, let alone kinases, and a search in PubMed for p38 MAPK or p38 kinase returns more than 36,000 publications, which is a higher number than some proteins listed in a review of the "top 10" most studied genes ^243^. By contrast, searches for the kinases Raf and Src return about 17,000 and 25,000 hits, respectively. In 2018, there were more than 2,000 publications that mention p38, and it is clearly impractical to summarize such a vast amount of literature. As might be surmised from the preceding commentary, the studies are on a wide range of topics; however, the publications are more concentrated in some areas than others. The role of the p38 pathway in cancers (>10,000) ^244– 246^, inflammation (>8,000) ^247– 249^, and infections (>3,600) ^250, 251^ was intensively studied. About 1,600 publications include the specific term "p38 inhibitor". This reflects the previously mentioned enormous interest of the pharmaceutical industry in developing p38 inhibitors to treat chronic inflammatory diseases, such as rheumatoid arthritis. Yet other publications report natural products that can activate or inhibit p38, with the ultimate aim of using them clinically ^252– 258^. In 2011, the European Commission approved Esbriet (pirfenidone), which was described as a p38γ inhibitor, for the treatment of idiopathic pulmonary fibrosis ^259^. However, when this drug was approved by the FDA in 2014 for treating the same disease, it was described as a compound that acts on multiple pathways. In 2008, there were 27 clinical trials listed testing the use of p38 inhibitors in inflammatory disease settings ^205^, while a search today for p38 inhibitors in clinicaltrials.gov returns 44 studies for conditions as diverse as pain, asthma, cognitive impairment, rheumatoid arthritis, cancer, myelodysplastic syndrome, and depression ( Table 5). This indicates that there remains clinical interest in targeting the pathway and that there is therefore a need for more specific inhibitors of each of the p38 group members and more basic research to fully understand how the pathway, especially how each member of the p38 family, is utilized and regulated.

**Table 5.** Clinical trials of p38 inhibitors.

Drug	Target	Condition or disease	Status	NCT number
ARRY-371797	p38	Ankylosing spondylitis	Phase 2	NCT00811499
ARRY-371797	p38	Dental pain	Phase 2	NCT00542035 NCT00663767
ARRY-371797	p38	Healthy	Phase 1	NCT00790049
ARRY-371797	p38	LMNA-related dilated cardiomyopathy	Phase 2	NCT02351856 NCT02057341
ARRY-371797	p38	Osteoarthritis of the knee	Phase 2	NCT01366014
ARRY-371798	p38	Rheumatoid arthritis	Phase 1	NCT00729209
ARRY-614	p38 and Tie2	Myelodysplastic syndromes	Phase 1	NCT01496495 NCT00916227
AZD7624	p38	Corticosteroid-resistant asthma	Phase 2	NCT02753764
BIRB 796 BS	p38	Healthy	Phase 1	NCT02211170
BMS-582949	p38α	Rheumatoid arthritis	Phase 2	NCT00605735
BMS-582949	p38α	Vascular diseases (atherosclerosis)	Phase 2	NCT00570752
CHF6297	p38α	Chronic obstructive pulmonary disease	Phase 1/2	NCT02815488
Losmapimod (GS856553)	p38α/β	Acute coronary syndrome	Phase 1/2/3	NCT01756495 NCT02145468 NCT00910962
Losmapimod (GS856553)	p38α/β	Chronic obstructive pulmonary disease	Phase 2	NCT00642148 NCT01541852
Losmapimod (GS856553)	p38α/β	Depressive disorder, major	Phase 2	NCT00976560 NCT00569062
Losmapimod (GS856553)	p38α/β	Glomerulosclerosis, focal segmental	Phase 2	NCT02000440
Losmapimod (GS856553)	p38α/β	Pain, neuropathic	Phase 2	NCT01110057 NCT00969059
LY3007113	p38	Metastatic cancer	Phase 1	NCT01463631
Neflamapimod (VX-745)	p38α	Alzheimer’s disease	Phase 2	NCT03402659 NCT02423200 NCT02423122
Neflamapimod (VX-745)	p38α	Dementia with Lewy bodies	Recruiting	NCT04001517
P38 inhibitor (4)	p38	Rheumatoid arthritis	Phase 2	NCT00303563 NCT00316771
PF-03715455	p38α	Asthma	Phase 2	NCT02219048
PF-03715455	p38α	Chronic obstructive pulmonary disease	Phase 2	NCT02366637
PF-03715455	p38α	Healthy	Phase 1	NCT01226693
PH-797804	p38α/β	Rheumatoid arthritis	Phase 2	NCT00383188 NCT00620685
Ralimetinib (LY2228820)	p38α/β	Adult glioblastoma	Phase 1/2	NCT02364206
Ralimetinib (LY2228820)	p38α/β	Advanced cancer	Phase 1	NCT01393990
Ralimetinib (LY2228820)	p38α/β	Epithelial ovarian cancer Fallopian tube cancer Primary peritoneal cancer	Phase 1/2	NCT01663857
Ralimetinib (LY2228820)	p38α/β	Postmenopausal metastatic breast cancer	Phase 2	NCT02322853
SB-681323	p38	Acute lung injury	Phase 2	NCT00996840
SB-681323	p38	Coronary heart disease	Phase 2	NCT00291902
SB-681323	p38	Chronic obstructive pulmonary disease	Phase 1/2	NCT00564746 NCT00144859
SB-681323	p38	Pain, neuropathic	Phase 2	NCT00390845
SB-681323	p38	Rheumatoid arthritis Inflammation	Phase 1/2	NCT00419809 NCT00439881 NCT00134693
Talmapimod (SCIO-469)	p38α	Bone marrow diseases Myelodysplastic syndromes Hematologic diseases Bone marrow neoplasms	Phase 2	NCT00113893
Talmapimod (SCIO-469)	p38α	Multiple myeloma	Phase 2	NCT00095680 NCT00087867
Talmapimod (SCIO-469)	p38α	Rheumatoid arthritis	Phase 2	NCT00043732 NCT00089921
VX-702	p38α	Rheumatoid arthritis	Phase 2	NCT00395577 NCT00205478

One consequence of the massive pharmaceutical effort over the last 20 years is a large number of very specific, well-tolerated, and readily bioavailable drugs that can enable such basic research. For example, one study using a boutique panel of kinase inhibitors was able to demonstrate that 11 potent and specific p38 inhibitors synergized with Smac-mimetic drugs to kill a subset of AML leukemias, providing the strongest evidence implicating p38 in Smac-mimetic-induced killing ^179^. Since several of these p38 inhibitors had already been clinically trialed, this presents an opportunity to fast-track such combinations into the clinic. In our opinion, it is likely that this is where the future of p38 research and p38 inhibitors lies, in revealing the intricate web of inter-connections and inter-dependencies of this core and central regulator of cell stress. We also believe that greater efforts to genetically assess the role of p38 and p38 isoforms in the pathophysiology of inflammatory and other diseases need to be made in order to push forward the clinical application of our burgeoning knowledge.
